# Comparative RNA-seq based transcriptome profiling of waterlogging response in cucumber hypocotyls reveals novel insights into the de novo adventitious root primordia initiation

**DOI:** 10.1186/s12870-017-1081-8

**Published:** 2017-07-26

**Authors:** Xuewen Xu, Minyang Chen, Jing Ji, Qiang Xu, Xiaohua Qi, Xuehao Chen

**Affiliations:** Department of horticulture, School of Horticulture and Plant Protection, Yangzhou University, 48 wenhui eastroad, Yangzhou, Jiangsu 225009 China

**Keywords:** Cucumber, Waterlogging, Adventitious root primordia, RNA-seq, Ethylene

## Abstract

**Background:**

Waterlogging is a serious abiotic stress to plant growth because it results in the decline in the supplement of oxygen to submerged tissues. Although cucumber (*Cucumis sativus* L.) is sensitive to waterlogging, its ability to generate adventitious roots (ARs) facilitates gas diffusion and increases plant survival when the oxygen concentration is decreased. To gain a better understanding of the molecular mechanisms that enable de novo AR primordia emergence upon waterlogging, the RNA sequencing-based transcriptomic responses of two contrasting cucumber genotypes, Zaoer-N (waterlogging tolerant) and Pepino (waterlogging sensitive), which differed in their abilities to form AR were compared.

**Results:**

More than 27,000 transcripts were detected in cucumber hypocotyls, from which 1494 and 1766 genes in ‘Zaoer-N’ and ‘Pepino’, respectively, were differentially expressed 2 days after waterlogging. The significant positive correlation between RNA sequencing data and a qPCR analysis indicated that the identified genes were credible. A comparative analysis revealed that genes functioning in carbohydrate mobilization, nitrate assimilation, hormone production and signaling pathways, transcription factors and cell division might contribute to the waterlogging-triggered AR primordia initiation. Ethylene was determined to be an important plant hormone responsible for the cucumber ARs initiation. Additionally, genes encoding cytochrome P450, ankyrin repeat-containing proteins and sulfite oxidases were determined as important in waterlogging acclimation.

**Conclusion:**

This research broadens our understanding of the mechanism underlying waterlogging-triggered ARs emergence, and provides valuable information for the breeding of cucumber with enhanced waterlogging tolerance.

**Electronic supplementary material:**

The online version of this article (doi:10.1186/s12870-017-1081-8) contains supplementary material, which is available to authorized users.

## Background

Waterlogging occurs in many regions worldwide because of poor drainage and/or excessive rainfall [1, 2]. The major plant symptoms caused by waterlogging include loss of plasma membrane integrity, leaf wilting, chlorosis and necrosis [3]. In addition, decreased growth rates and yields have been attributed to the lack of available oxygen required to support aerobic respiration, and the associated increase in temperature causes more damage [4]. To adapt to a waterlogging environment, some tolerant plants have evolved morphological adaptations, such as the formation of adventitious roots (ARs), formation of aerenchyma in existing tissues, and elongation of shoots, all of which improve the ability to supply oxygen to hypoxic tissues [5, 6].

ARs production is an important adaptive trait of waterlogging tolerance [7], because it allows the submerged tissues to obtain oxygen directly from the air. This characteristic has been reported in rice [8], *Rumex* [9], barley [10], maize [11], soybean [12] and *Solanum dulcamara* [6]. In most species, the waterlogging-induced ARs also contain highly porous tissues, such as extensive aerenchyma. AR formation is usually divided into four key steps: (i) cell dedifferentiation, (ii) cell division, (iii) AR primordia outgrowth and (iv) AR elongation [13]. In rice and *S. dulcamara*, AR primordia formed at the nodes as a part of the normal developmental program and grow at a very slow rate, resulting in larger root initials as the nodes age [14]. These dormant primordia become activated and emerge from the stem node within 8–10 h of partial waterlogging [15]. In case of deep-water rice, ethylene is released upon waterlogging, inducing the formation of the reactive oxygen species (ROS). This results in the death of cell in the epidermal cell layer that covers the AR primordia at the nodes, thereby facilitating their emergence [14, 16]. A global gene expression study in *S. dulcamara* indicated that multiple hormones signaling pathways are likely to be involved by flooding and, therefore, may activate the dormant AR primordia [6]. However, in most dicotyledons, such as cucumber and tomato, no cells are specified to form ARs in hypocotyls or stems before induction [17]. In this case, continuous cell division, growth, and differentiation are essential to produce the AR primordia inside the hypocotyls [18]. To date, the mechanism responsible for waterlogging’s effects on de novo AR primordia initiation and further development remain unclear.

Cucumber, *Cucumis sativus* L., is an economically important vegetable crop [2]. Cucumber has a shallow root system and strict oxygen requirement [19, 20]. Thus, cucumber is generally considered as waterlogging sensitive, and is easily affected by heavy rain and subsequent periods of soil waterlogging [19]. In our screening of germplasm, we identified a cucumber landrace, ‘Zaoer-N’ that exhibited a high level of waterlogging tolerance [21]. In greenhouse waterlogging experiments, Zaoer-N produced large numbers of ARs on its hypocotyl, while almost no ARs were generated in the waterlogging-sensitive line Pepino. To further our understanding of the molecular mechanisms that enable de novo AR primordia emergence and to improve gas exchange upon an abiotic stress trigger, we analyzed the transcriptomic changes of these two contrasting cucumber genotypes that differed in waterlogging tolerance and in ARs formation capabilities [6]. Our findings will allow the identification of important genes involved in waterlogging-trigged AR formation in cucumber hypocotyls, resulting in important molecular resources for the further breeding of cucumber with an enhanced tolerance to waterlogging.

## Methods

### Plant materials and stress conditions

The waterlogging tolerant line Zaoer-N is a cucumber landrace originally collected from South China, and has been maintained in our laboratory for more than 20 years. The waterlogging sensitive ‘Pepino’ is a North American processing market-type cucumber cultivar, which was kindly provided by U.S. National Plant Germplasm System (https://npgsweb.ars-grin.gov/gringlobal/search.aspx?). ‘Zaoer-N’ and ‘Pepino’ were grown in 8-cm-wide pots containing vermiculite, peat and perlite (1:3:1, *v*/v/v) in a greenhouse with a relative humidity ranging from 70 to 85% and 28 °C/20 °C (14 h/10 h) day/night temperature. Upon the emergence of the third true leaves (21 d after germination), the seedlings were transferred into plastic containers filled with water (pH 7.03, dissolved oxygen level 7.17 mg/L and electrical conductivity of 0.34 dS m^−1^) to the top of hypocotyls (~4 cm above the soil surface with a soil redox potential of 272 ± 4.5 mV). Water was kept constant throughout the experiment. For control treatments, plants were placed in containers with no water. Hypocotyls (below the water surface) were harvested from the ‘Zaoer-N’ and ‘Pepino’ seedlings 2 d after treatment, frozen in liquid nitrogen, ground into powder and stored at −80 °C. There were three biological replicates for each sample, which consisted of 15 hypocotyls from 15 seedlings of the same line.

### Abscisic acid (ABA), indole-3-acetic acid (IAA) and ethylene determinations

The endogenous ABAand IAA contents were extracted and analyzed according to the methods of Großkinsky et al. [22] and Kim et al. [12]. Briefly, 0.8 g of frozen powder were extracted twice with 4 mL of extraction solution. After shaken for 30 min at 4 °C, dichloromethane (8 mL) was added, and the mixture was further shaken at 4 °C for 30 min and centrifuged in an Eppendorf 5415D centrifuge (Eppendorf, Hamburg, Germany) for 5 min at 13,000 rpm. The lower organic phase was protected from light, dried under nitrogen, dissolved in methane acid and filtered using a 0.45-μm membrane. The filtrated extract was then subjected to HPLC–MS/MS analysis using a reversed-phase ZORBAX SB-C18 (Agilent Technologies, Santa Clara, CA, USA) column (150 × 2.1 mm; 3.5 μm particle size). The mobile phase A solvent consisted of 0.1% methanoic acid /methanol, and the mobile phase B solvent consisted of 0.1% methanoic acid/ultrapure water.

The fresh hypocotyl samples used in the ethylene analysis were from the same batches (before being frozen) used for the RNA-seq experiment. The washed samples were kept in 30-mL dark glass bottles for 30 min. Then, 1 mL gas samples were injected into a gas chromatograph device equipped with a TRB-5 capillary column. Injection, detector and heat chamber temperatures were set at 150 °C, 150 °C and 100 °C, respectively. The gas chromatograph was calibrated with standard ethylene to determine the exact retention time before each endogenous ethylene determination.

### RNA extraction, library construction and Illumina sequencing

Total RNA was extracted from the hypocotyls of treatment and control seedlings of both lines using TRIzol reagent (Invitrogen, Carlsbad, CA, USA). We confirmed the RNA integrity using the 2100 Bioanalyzer (Agilent Technologies, USA). We measured the RNA concentration in a Qubit 2.0 fluorometer by the Qubit RNA Assay Kit (Life Technologies, Carlsbad, CA, USA). We prepared the Libraries from 100 ng of total RNA using an Illumina TruSeq RNA Sample Prep Kit (San Diego, CA, USA) following the manufacturer’s protocol. In total, 12 libraries were sequenced using the Illumina Hiseq 2500 platform (San Diego, CA, USA). We pre-processed the raw reads by our in-house quality control pipeline. The clean sequence reads were then blasted to the 9930 reference genome (http://www.icugi.org/cgi-bin/ICuGI/index.cgi), and then, the mRNA abundance levels of the unigenes identified using TopHat v2.0.9 and Cufflinks [23] were normalized by the Fragments Per Kilobase of exon model per Million mapped reads (FPKM) [24],and the log2 fold changes between two samples were tested statistically to determine whether an individual gene’s expression was altered significantly. We used the criteria of false discovery rate (FDR) < 0.01 and fold changes <0.5 or >2.0 (< −1 or >1 in log2 ratio value) to identify the differentially expressed genes (DEGs).

### Quantitative PCR (qPCR) analysis

To validate the RNA-seq results, a subset of DEGs were verified by qPCR. An independent set of samples was harvested at 48 h after waterlogging, and a corresponding control was used for the expression analysis. Sequences for each gene from the 9930 genome database (http://www.icugi.org/cgi-bin/ICuGI/index.cgi) were used to design primers with the online Primer 3 tool (http://bioinfo.ut.ee/primer3-0.4.0/; Additional file 1: Table S1). The expression levels of 15 cucumber genes were tested using the RealMasterMix (SYBR Green) kit (Tiangen, China) following the manufacturer’s protocol with an iQ™ 5 Multicolor real-time PCR detection system (Bio-RAD, USA). To verify the presence of specific products, melting curve analyses of amplification products were executed following each PCR reaction.

## Results and discussion

### ‘Zaoer-N’ and ‘Pepino’ have contrasting responses, reflecting their waterlogging tolerance difference

**Fig. 1:**
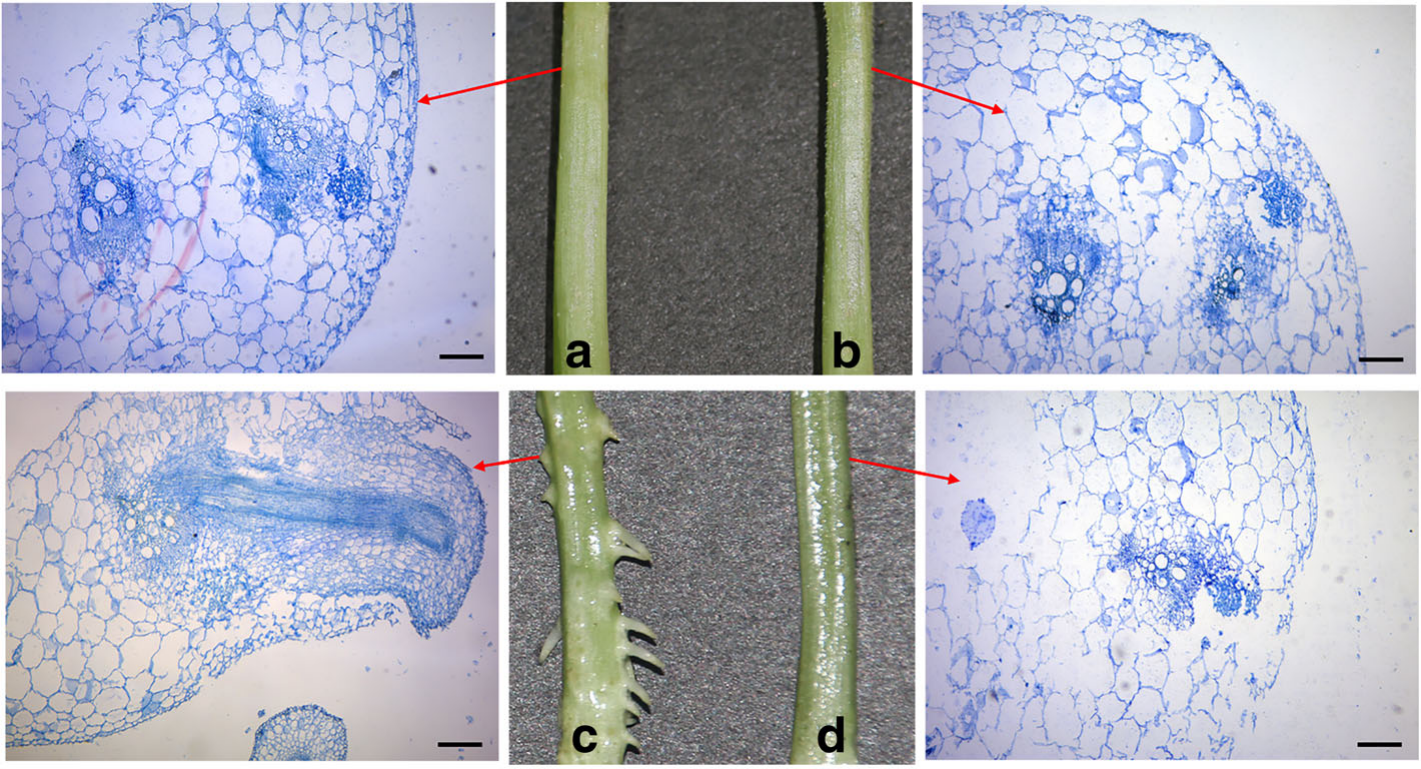
Adventitious root formation on hypocotyls of cucumber seedlings. **a** Zaoer-N control; **b** Pepino control; **c** Zaoer-N waterlogging; **d** Pepino waterlogging. The water level was kept at about 4 cm (to the base of the first true leaves) above the soil for 2 days and then removed for photography

Extended waterlogging periods result in the survival of selected cultivars or species [25]. The waterlogging-tolerant line ‘Zaoer-N’ is a landrace collected from South China where crop plants are subjected to frequent waterlogging or flooding events. The high frequency and long duration of waterlogging stress experienced by ‘Zaoer-N’ has led to a strongly selected for plants with greater levels of low oxygen and waterlogging tolerance compared with those of ‘Pepino’. A visual contrasting survival strategy adopted during the seedling stage is the capacity for AR formation in hypocotyls, which is shown in Fig. 1 and Additional file 2: Fig. S1. No AR primordia were observed in either genotype before the waterlogging treatment (Fig. 1a, b), which was different from previous reports on rice and *S. dulcamara* [6, 26]. In these species, AR primordia were constitutively present on the stem or internodes before waterlogging. Here, AR primordia were visible only on the hypocotyl surfaces of ‘Zaoer-N’ 2 days after waterlogging, compared with control plants and waterlogged ‘Pepino’ (Fig. 1c, d). Seven days after treatment, the average AR numbers protruding from the basal parts of ‘Zaoer-N’ and ‘Pepino’ hypocotyls were 30.4 and 2.8, respectively (Additional file 2: Fig. S1). To elucidate the molecular changes that occurred during AR emergence in cucumber, plants were waterlogged for 2 days and the hypocotyls tissues were then dissected separately from ‘Zaoer-N’ and ‘Pepino’ for RNA-seq analysis.

### Waterlogging causes extensive transcriptomic reprogramming

**Fig. 2:**
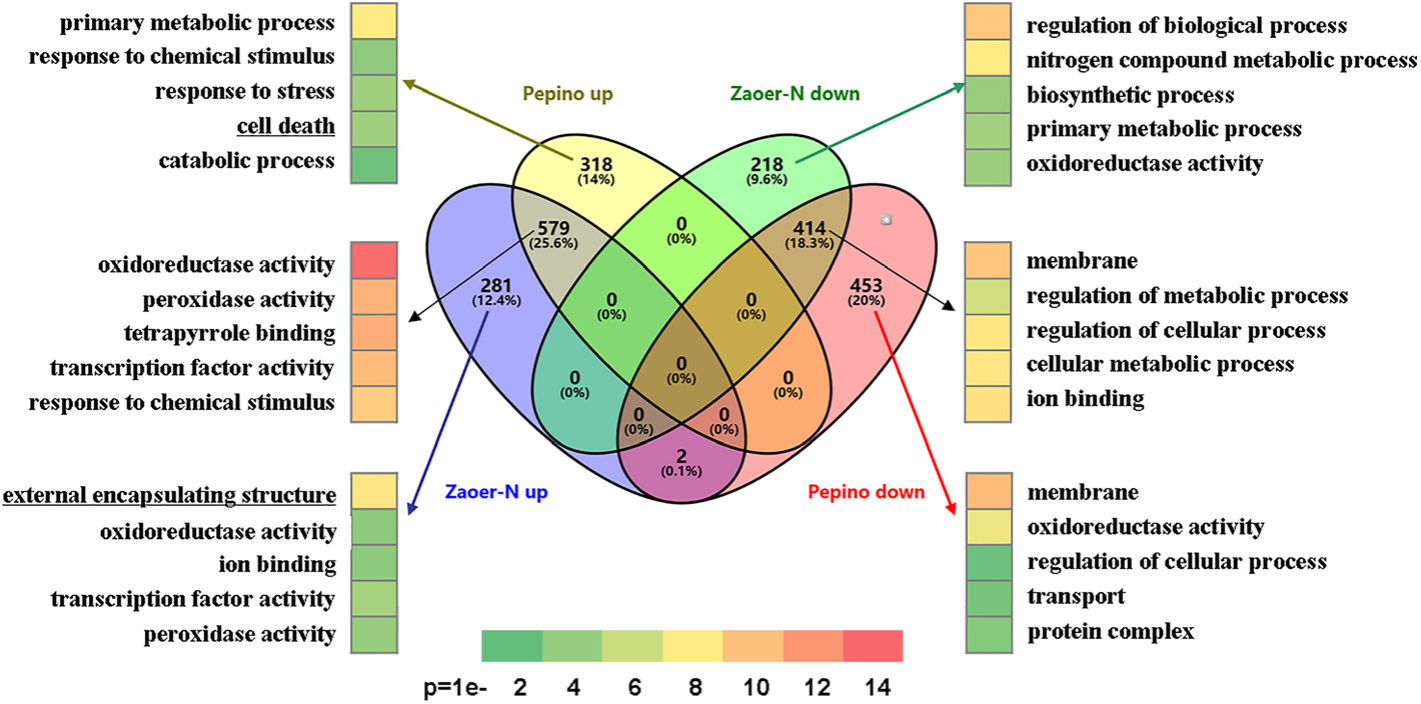
Venn diagram of all genes expressed in hypocotyls of Zaoer-N and Pepino upon waterlogging (false discovery rate ≤ 0.01 and fold change ≥2) and their respective top five most significantly enriched Gene Ontology (GO) terms

**Fig. 3:**
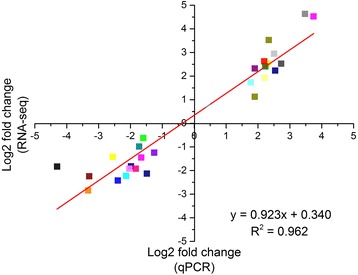
Comparison of protein folds change levels measured by RNA-seq and quantitative real-time reverse transcription-PCR (qPCR) assays. The gene fold changes log2-values (Y-axis) were plotted against the qPCR fold changes log2-values (X-axis). The cucumber β-actin gene (GenBank AB010922) was used as an internal control to normalize the expression data. Each value denotes the mean relative level of expression of three biological replicates

After removing the unknown (the proportion of undetermined bases >10%), low-quality and adaptor-containing reads, at least 28.3 million clean reads were obtained for each sample (Table 1). The clean reads were subsequently mapped to the cucumber 9930 genome assembly Ver. 2 [27]. In total, the expression of 27,058 transcripts was detected. Approximately 87% of the clean reads were mapped to the reference genome, with more than 83% of them being uniquely mapped (Table 1). Using a fold change ≥2 and FDR ≤ 0.01 as cut-offs, 1494 (Additional file 3: Table S2) and 1766 (Additional file 4: Table S3) DEGs were identified in the pairwise comparisons of ‘Zaoer-N’ waterlogging vs. ‘Zaoer-N’ control and ‘Pepino’ waterlogging vs. ‘Pepino’ control, respectively, indicating that a large number of genes were waterlogging responsive. In total, 993 DEGs (579 up-regulated and 414 down-regulated in abundance) were shared between the two lines. Additionally, 499 DEGs (281 up-regulated and 218 down-regulated) were only identified in ‘Zaoer-N’, and 771 DEGs (318 up-regulated and 453 down-regulated) were only identified in ‘Pepino’. Only two DEGs (*Csa5G153010* and *Csa5G305760*) showed opposite regulatory states in the two lines (Fig. 2). To validate the RNA-seq data, 15 randomly selected DEGs were subjected to qPCR analyses. As shown in Fig. 3, there was a strong positive correlation (two tailed, R^2^ = 0.96) between the RNA-seq data and qPCR data, indicating that the RNA-seq data were credible.

**Table 1:** Mapping results of RNA sequencing reads of the cucumber ‘Zaoer-N’ and ‘Pepino’ 48 h after waterlogging (WL) treatment and relative non-waterlogged conditions (CK)

Sample ID	Total reads	Mapped reads	Uniquely mapped reads	Number of mapped genes
Pepino CK_1	33,225,482	29,848,764	28,965,262	19,723
Pepino CK_2	46,772,956	41,794,366	40,689,862	21,056
Pepino CK_3	37,596,070	33,665,760	32,653,010	19,991
Pepino WL_1	36,258,376	32,161,091	29,798,229	20,038
Pepino WL_2	36,982,414	32,480,793	30,615,432	20,052
Pepino WL_3	41,844,460	35,644,650	29,096,668	20,247
Zaoer-N CK_1	43,620,302	37,461,442	34,791,432	20,050
Zaoer-N CK_2	35,427,694	30,387,272	29,598,977	19,870
Zaoer-N CK_3	39,279,546	34,107,693	33,275,553	20,013
Zaoer-N WL_1	42,364,332	36,674,382	35,372,191	20,346
Zaoer-N WL_2	28,383,702	2,4680,987	23,953,530	19,918
Zaoer-N WL_3	36,077,522	31,208,030	30,126,104	20,145
Average	38,152,738	33,342,936	31,578,021	20,121

### Functional annotation of waterlogging-responsive DEGs

To dissect the processes taking place during the AR primordia emergence under waterlogging conditions, we peformed a gene ontology (GO) enrichment analysis. The top five most significantly enriched GO terms are shown in Fig. 2. The GO terms ‘response to chemical stimulus’ and ‘oxidoreductase activity’ were highly enriched in the DEGs that were commonly up-regulated in ‘Zaoer-N’ and ‘Pepino’, whereas ‘regulation of cellular process’ and ‘regulation of metabolic process’ were highly enriched in the DEGs that were commonly down-regulated in the two lines. The physical challenge of oxygen deprivation under waterlogging is reflected in these metabolic changes, in which both lines appear to activate reserve mobilization and down-regulate energy-consuming processes. The GO term ‘peroxidase activity’ was highly enriched in the DEGs that were specifically up-regulated in ‘Zaoer-N’, but ‘cell death’ was dramatically enriched in those specifically up-regulated in ‘Pepino’. The impairment of photosynthesis under waterlogging generally leads to metabolic imbalances and, thus, the production of ROS and toxic byproducts [28]. The overrepresentation of GO terms associated with peroxidase activity in ‘Zaoer-N’ suggested enhanced ROS scavenging during AR emergence. ROS at lower concentrations may act as signaling molecules involved in the acclimation to environmental stress, but high levels of these compounds are also harmful to plant cells, which was reflected in the additional activation of the ‘cell death’ GO term in ‘Pepino’ [29]. The GO term ‘external encapsulating structure’, which refers to a structure that lies outside the plasma membrane and surrounds the entire cell, was also highly enriched in the genes that were specifically up-regulated in ‘Zaoer-N’ [30]. This enrichment suggested that AR emergence in ‘Zaoer-N’ hypocotyls was associated with cell reconstruction. Furthermore, there was a strong overrepresentation of the GO term ‘nitrogen compound metabolic process’ in the down-regulated DEGs of ‘Pepino’. The beneficial effects of the metabolism of nitrogen-containing compounds in plants under waterlogging conditions involve NAD^+^ regeneration, ATP synthesis and cytosolic acidification [31]. Compromising the expression of genes involved in the nitrogen compound-related metabolic processes could further block the ATP supply and reduce the cytoplasmic pH in ‘Pepino’ hypocotyls.

### Differential regulation of genes associated with energy production

Because oxygen-dependent mitochondrial respiration is greatly limited under waterlogging conditions, the acceleration of carbohydrate metabolism is functionally conserved among both sensitive and tolerant plants, and is critical for plant survival [32]. As expected, we found that several DEGs, such as *glucose-6-phosphate 1-epimerase* (*Csa4G050850*), *pyruvate kinase* (*Csa5G580610* and *Csa6G449830*), *6-phosphogluconate dehydrogenase* (*Csa5G642720*), *triose phosphate isomerase* (*Csa4G598000*) and *phosphofructokinase* (*Csa4G664520*), which are involved in glycolysis, and *alcohol dehydrogenase* (*ADH*; *Csa7G320050*) and *pyruvate decarboxylase* (*PDC*; *Csa3G730980* and *Csa6G518930*), which are involved in ethanol fermentation were significantly accumulated in both ‘Zaoer-N’ and ‘Pepino’, suggesting the common regulation of energy generation in waterlogged cucumber hypocotyls.

The maintenance of the adenylated energy charge is dependent on the availability of substrates for the glycolytic and fermentative pathway, such as glucose, fructose or pyruvate. Sucrose synthase is a key enzyme for the hydrolysis of sucrose, and it plays a important role in providing an adequate sugar supply during waterlogging [33]. Ricard et al. [34] found that maize *sucrose synthase* mutants are less tolerant to oxygen deficits than the wild-type. Wang et al. [35] reported that the overexpression of *sucrose synthase* in cucumber confers tolerance to hypoxic conditions. Here, the gene (*Csa1G597800*) encoding sucrose synthase was highly up-regulated in tolerant ‘Zaoer-N’ but remained unchanged in susceptible ‘Pepino’. In addition to sucrose synthase, genes encoding aspartate aminotransferase (EC2.6.1.1, *Csa3G889160*) and alanine aminotransferase (EC2.6.1.2, *Csa3G646610*), which are involved in pyruvate regeneration by affecting the amount of oxaloacetic acid [36], were also induced at higher levels in hypocotyl cells of ‘Zaoer-N’ than ‘Pepino’ seedlings under waterlogging conditions. Consequently, ‘Zaoer-N’, which sustained adequate supplies of readily useable sugars and additional pyruvate generation from amino acid metabolism, had a greater energy state and chance of survival than ‘Pepino’, which was lacking in these areas.

Stoimenova et al. [37] found that plant mitochondria are capable of anaerobic ATP synthesis with NADH and NADPH as electron donors and nitrite as a terminal electron acceptor, replacing oxygen in the respiratory chain. Nitrate is the main nitrogen source for most plants, and its application increased the survival of tomato and *Hevea brasiliensis* subjected to waterlogging [31, 38]. Plants take up nitrate from the soil using transporters in the plasma membrane of root epidermal and cortical cells. In waterlogged cucumber hypocotyls, 11 and 12 DEGs encoding nitrate transporters (NRTs) were identified respectively in ‘Zaoer-N’ and ‘Pepino’, emphasizing the roles of nitrate in waterlogging acclimation. Because seven of the 11 NRTs in ‘Zaoer-N’ were up-regulated, while eight of the 12 NRTs in ‘Pepino’ were down-regulated (Table 2), nitrate may be assimilated to a greater degree in waterlogged ‘Zaoer-N’ hypocotyls (Table 2). The extra assimilated nitrate could be used as NADH acceptors, providing an alternative to fermentation during waterlogging. The denitrification has some advantages for survival and AR emergence if it operates in conjunction with a means of removing the toxic byproduct nitric oxide (NO), an important factor in cell death induction [39]. The non-symbiotic hemoglobins (nsHBs) could function in this regard. Perazzolli et al. [40] found that *Arabidopsis AtnsHB1*, scavenges NO through the production of S-nitrosohemoglobin and reduces NO emission under hypoxic stress, indicating its role in NO detoxification. Here, we observed that two *nsHBs* (*Csa2G238880* and *Csa2G375770*) were commonly upregulated in both genotypes and their expression levels were greater higher in the waterlogged ‘Zaoer-N’ hypocotyls (Table 2). Thus, ‘Zaoer-N’, with its efficient metabolic mechanisms associated with carbohydrate mobilization and nitrogen assimilation, may be more suited to face oxygen deprivation and generate AR primordia. This speculation is in accordance with our previous measurements in which a 2-d waterlogging treatment led to greater increases in PDC and ADH activities, as well as ethanol concentrations, in the hypocotyls of ‘Zaoer-N’ than in those of ‘Pepino’ [2]. However, additional biochemical testing is needed to confirm the importance of nitrogen assimilation on plant survival under waterlogging stress.

**Table 2:** Comparisons of the expression levels of representative nitrate transporter genes and non-symbiotic hemoglobin genes involved in nitrate assimilation and NO scavenging. ‘ND’ represents not detected

Gene ID	Z WL vs Control		P WL vs Control		Functional annotation
	Log2FC	FDR	Log2FC	FDR	
Csa1G008530	−1.54	1.46E-08	−1.36	2.78E-04	nitrate transporter 2.11
Csa2G374640	−2.81	2.39E-06	−1.78	1.58E-03	nitrate transporter 1.2-like
Csa4G007610	−1.71	1.30E-07	−1.51	1.93E-05	nitrate transporter 6.1
Csa3G027720	2.61	1.95E-04	2.48	5.24E-18	nitrate transporter 1.1-like
Csa3G134770	−2.15	2.29E-04	1.39	1.29E-03	nitrate transporter 5.2-like
Csa5G161290	7.05	1.10E-06	7.42	3.41E-15	nitrate transporter 6.3-like
Csa1G324370	ND	ND	−1.08	8.85E-04	nitrate transporter 6.2-like
Csa2G172510	ND	ND	−1.07	1.85E-05	nitrate transporter 5.2
Csa2G416080	ND	ND	−1.52	1.55E-04	nitrate transporter 6.4
Csa3G112250	ND	ND	−2.11	4.16E-05	nitrate transporter 4.3
Csa5G647280	ND	ND	−1.43	8.19E-05	nitrate transporter 4.4
Csa3G904070	ND	ND	1.04	1.59 E-03	nitrate transporter 7.3-like
Csa3G134760	2.45	6.97E-04	ND	ND	nitrate transporter 5.3
Csa6G037500	1.23	1.53E-05	ND	ND	nitrate transporter 3.1
Csa6G404120	1.16	7.62 E-03	ND	ND	nitrate transporter 4.6-like
Csa5G219390	1.24	2.96E-05	ND	ND	nitrate transporter 5.1
Csa6G187970	1.31	3.13E-06	ND	ND	nitrate transporter Y 8.1
Csa2G238880	3.38	2.43E-20	2.45	2.27E-07	non-symbiotic hemoglobin 1
Csa2G375770	3.29	1.81E-06	2.83	1.51E-27	non-symbiotic hemoglobin 3

### Hormonal regulation of AR emergence in waterlogged cucumber

**Fig. 4:**
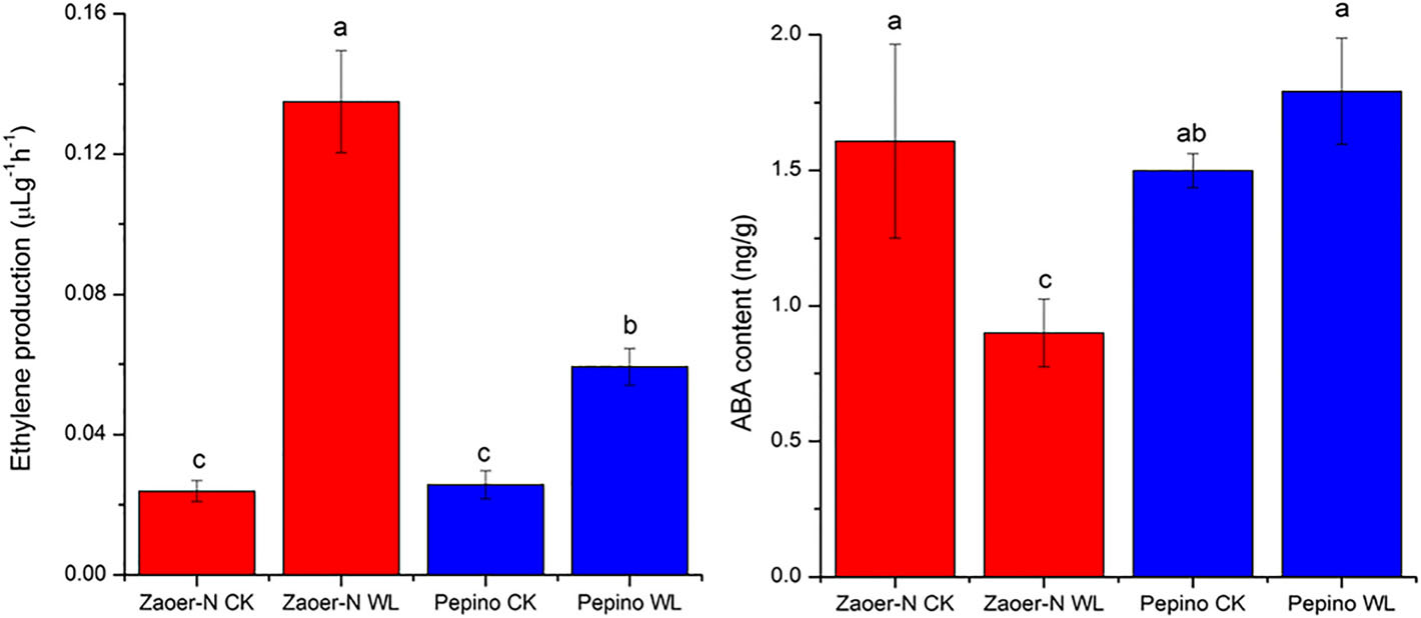
Ethylene production (*left*) and endogenous ABA (*right*) in Zaoer-N and Pepino 2 days after waterlogging treatment. ‘WL’ and ‘CK’ represent waterlogging treatment and control. Data is the average ± standard error (*n* = 3). Means with the same lowercase letter do not significantly differ by the least significant difference (LSD) test at *p* ≤ 0.05 with a completely randomized design

Ethylene is a major hormone of waterlogging-induced AR emergence [41]. In the submergence-tolerant species *S. dulcamara*, submergence for 24 h induces a significantly increase in ethylene emission that in turn promotes the activation of AR primordia, which can be delayed by a pretreatment with the ethylene-perception inhibitor 1-methylcyclopropene (1-MCP) [6]. Accordingly, we measured the endogenous ethylene production of the waterlogged hypocotyls. Increase in ethylene release 48 h after waterlogging of ~5.7-fold and 2.3-fold were observed in ‘Zaoer-N’ and ‘Pepino’, respectively (Fig. 4). In our previous study, we found that the AR primordia generated on ‘Zaoer-N’ hypocotyls after waterlogging were significantly inhibited when pretreated with 100 mg/L 1-MCP as an inhibitor of ethylene action [42], indicating the importance of ethylene in waterlogging-triggered cucumber AR production. Ethylene is biosynthesized from methionine, and it is produced by the activation of 1-aminocyclopropane-1-carboxylicacid synthase (ACS) and ACC oxidase (ACO) [43]. The transcriptome-wide analysis showed that transcripts of an *ACS* (*Csa4G049610*) and three *ACO* (*Csa2G000520*, *Csa4G056660* and *Csa6G421630*) accumulated in both lines, but the gene inductions in ‘Zaoer-N’ were greater, which was in line with the observed difference in ethylene production. Ivanchenko et al. [44] also found that enhanced ethylene synthesis promotes the initiation of *Arabidopsis* lateral root primordia.

The environmental stress-associated hormone ABA is a known inhibitor of lateral roots development that acts at the post-emergence stage [12, 45]. Contrary to the case of ethylene, ABA responses were down-regulated upon waterlogging according to our transcriptome analysis. Two DEGs (*Csa4G064690* and *Csa6G523440*) encoding 9-cis epoxycarotenoid dioxygenase (NCED) were especially repressed in ‘Zaoer-N’. NCED catalyzes the cis-violaxanthin to xanthoxin, a direct precursor of ABA synthesis [46] and, therefore, may contribute to a reduction in ABA, providing conditions favorable for AR induction. A strong decrease in *carotenoid cleavage dioxygenase* (*Csa4G056640*), which may also include NCEDs, was observed in ‘Zaoer-N’ only. To confirm this, the endogenous ABA content 2 days after waterlogging treatment were determined, and the ABA level in waterlogged ‘Zaoer-N’ hypocotyls decreased approximately 63.9% in comparison with the control, whereas there was no significant difference in ‘Pepino’ (Fig. 4). This indicates that the maintenance of ABA levels could facilitate AR growth suppression in ‘Pepino’. The decrease in the ABA levels in ‘Zaoer-N’ may result from the ethylene accumulation, which has been reported in plant species such as soybean [12] and *Rumex palustris* [47].

Based on functional annotations, there were 29 (17 down-regulated and 11 up-regulated) and 30 (17 down-regulated and 13 up-regulated) DEGs in ‘Zaoer-N’ and ‘Pepino’, respectively, that were involved in auxin metabolism (Table 3). These included transcripts encoding IAA-amido synthetases (GH3s), auxin efflux carrier components (PIN), indole-3-pyruvate monooxygenase YUCCA (YUC), auxin response factors (ARF) and auxin-induced or -responsive proteins. *GH3* is of special interest because it is an adenylate-forming enzyme in plants that conjugates amino acids to auxin, providing a negative feedback loop to control auxin homoeostasis [48]. The overexpression mutants GH3–6/DFL1 [49] and GH3–2/YDK1 [50] display dwarf phenotypes consistent with decreased levels of free auxin in these plants. A strong induction of *GH3s* (*Csa6G493310*, *Csa4G007100* and *Csa3G198490*) was found in both genotypes under waterlogging stress, indicating that the mechanisms associated with auxin storage were important negative regulatory genes for AR initiation in cucumber hypocotyls. Auxin directs plant morphogenesis through the differential accumulation within tissues, which depends largely on the activity of PIN proteins that mediate the auxin efflux from cells and thus directional cell-to-cell transport [51]. The Arabidopsis triple mutant *PIN1PIN*3*PIN*4 displays defects in primary root development [52]. The PIN2-RNA interference lines in *S. dulcamara* delayed AR emergence upon flooding [6]. Among the four *PINs* (*Csa1G025070*, *Csa1G042820*, *Csa4G430820* and *Csa5G576590*) identified in our transcriptome data, *Csa1G025070* and *Csa1G042820* were commonly down-regulated in both genotypes, while *Csa4G430820* was specifically accumulated in ‘Zaoer-N’, and *Csa5G576590* was specifically decreased in ‘Pepino’. We hypothesized that the up-regulation of *PIN* (*Csa4G430820*) in ‘Zaoer-N’ may contribute to basipetal auxin transport to the hypocotyl meristem, thereby, promoting AR primordia emergence. Additionally, the crucial gene *YUC4* (*Csa2G379350*) for auxin biosynthesis was down-regulated in the hypocotyls of ‘Pepino’. There are 11 YUC family genes in the *Arabidopsis* genome [53]. The overexpression of each *YUC* in transgenic *Arabidopsis* plants leads to auxin overproduction, but the disruption of these *YUC* genes leads to defects in embryogenesis, seedling growth, flower development, and vascular pattern formation [54, 55]. The down-regulation of *YUC4* in ‘Pepino’ may at least partially account for the compromised ability to induce AR. However, not all auxin-induced genes act as positive regulators during AR formation, and many of them were also found to be down-regulated in ‘Zaoer-N’, especially those genes encoding ARFs (*Csa7G329330*, *Csa6G518210* and *Csa6G291920*) and auxin transporters (Table 3). de Jong et al. [56] found that *SlARF9* negatively controls cell division during early tomato fruit growth. A transgenic line that over-expressed *SlARF9* formed smaller fruits than wild-type, whereas a transgenic line that had a reduced *SlARF9* mRNA expression level showed the opposite phenotype. Wang et al. [57] found a large number of genes involved in auxin transport were downregulated during indole-3-butyric acid-induced AR formation in softwood cuttings of *Catalpa bungei*. An in-depth functional analysis of these downregulated auxin metabolism-related DEGs is required to further reveal their exact roles in AR formation.

**Table 3:** Comparisons of the expression levels of representative differentially expressed genes involved in auxin metabolism. ‘ND’ represents not detected

Gene ID	Z WL vs Control		P WL vs Control		Functional annotation
	Log2FC	FDR	Log2FC	FDR	
Csa3G198490	ND	ND	1.84	2.45 E-03	Indole-3-acetic acid-amido synthetase GH3.1
Csa4G007100	2.53	1.07E-11	1.33	1.63 E-03	Indole-3-acetic acid-amido synthetase GH3.7
Csa6G493310	2.79	1.86E-08	ND	ND	indole-3-acetic acid-amido synthetase GH3.1
Csa1G025070	−1.24	7.86 E-06	−1.46	5.93E-08	Auxin efflux carrier component 1
Csa1G042820	−1.21	3.22 E-06	−1.05	3.58E-05	Auxin efflux carrier component 1
Csa4G430820	1.57	7.53 E-04	ND	ND	Auxin efflux carrier component 1c
Csa5G576590	ND	ND	−1.46	4.09E-08	Auxin efflux carrier component 3
Csa1G051690	1.99	4.17 E-03	ND	ND	Indole-3-acetic acid-induced protein ARG7
Csa5G534970	ND	ND	−1.89	9.77 E-04	Indole-3-acetic acid-induced protein ARG5
Csa6G452710	6.07	2.51 E-08	5.07	2.38E-17	Indole-3-acetic acid-induced protein ARG7
Csa1G207820	−1.51	4.6 E-05	−1.17	9.23E-05	Auxin-responsive protein IAA13
Csa1G397130	ND	ND	3.18	9.69E-06	Auxin-responsive protein IAA16
Csa2G059200	ND	ND	1.75	2.85 E-04	Auxin-responsive protein IAA33
Csa2G170820	ND	ND	−1.79	1.11E-11	Auxin-responsive protein IAA13
Csa2G200420	−1.42	7.4 E-03	ND	ND	Auxin-responsive protein
Csa3G134550	ND	ND	−1.25	4.61E-05	Auxin-responsive protein IAA13
Csa3G143580	−1.09	3.23 E-03	ND	ND	Auxin-responsive protein IAA4
Csa3G877650	−1.22	7.92E-04	ND	ND	Auxin-responsive protein IAA29
Csa6G497220	1.11	1.13 E-04	ND	ND	Auxin-responsive protein IAA9
Csa2G258720	−2.56	4.09 E-03	ND	ND	Auxin-induced protein 6B
Csa2G258760	2.62	7.53 E-05	ND	ND	Auxin-induced protein X10A
Csa2G379350	ND	ND	−1.85	4.50 E-06	Indole-3-pyruvate monooxygenase YUCCA4
Csa3G118740	−1.09	2.65 E-05	−1.42	8.34E-08	Auxin-induced protein X15
Csa3G171820	−1.03	1.83 E-03	ND	ND	Auxin-induced protein
Csa3G821040	ND	ND	3.89	3.09E-06	Auxin-regulated gene
Csa3G866530	ND	ND	2.64	2.66 E-04	Auxin-induced protein 6B
Csa3G883020	ND	ND	2.34	1.07 E-04	Auxin-induced protein 6B
Csa4G308640	ND	ND	−1.20	3.00E-06	Auxin transporter-like protein 4
Csa4G433470	ND	ND	3.85	1.35E-06	Auxin-regulated gene I
Csa4G556180	−1.55	5.03 E-04	−2.02	3.41E-09	Auxin-induced protein 6B OS
Csa5G623890	ND	ND	1.52	1.10 E-04	Auxin-regulated gene
Csa6G092560	2.32	1.22E-10	2.08	1.54E-16	Auxin-induced protein 15A
Csa6G291920	−1.11	3.99 E-06	−1.10	1.72E-05	Auxin response factor 4
Csa6G518000	−1.44	3.09 E-03	ND	ND	Auxin-repressed 12.5 kDa protein
Csa6G518210	−1.07	1.81 E-05	−1.07	6.00E-05	Auxin response factor 3
Csa7G008430	1.19	4.21 E-04	ND	ND	Auxin-induced protein 15A
Csa7G009020	−2.40	4.67 E-05	ND	ND	Auxin-induced protein X10A
Csa7G009150	2.50	1.39 E-05	2.54	2.40 E-04	Auxin-induced protein 10A5
Csa7G010800	ND	ND	−1.30	1.71E-06	Auxin transporter-like protein 5
Csa7G329330	−1.20	5.00 E-06	−1.33	2.39 E-03	Auxin response factor 9
Csa7G378520	−1.34	2.39 E-03	ND	ND	Auxin-induced protein AUX28
Csa7G448680	2.92	6.39E-15	1.68	4.30E-05	Auxin responsive protein

### Responses of transcription factors (TFs) to waterlogging

**Fig. 5:**
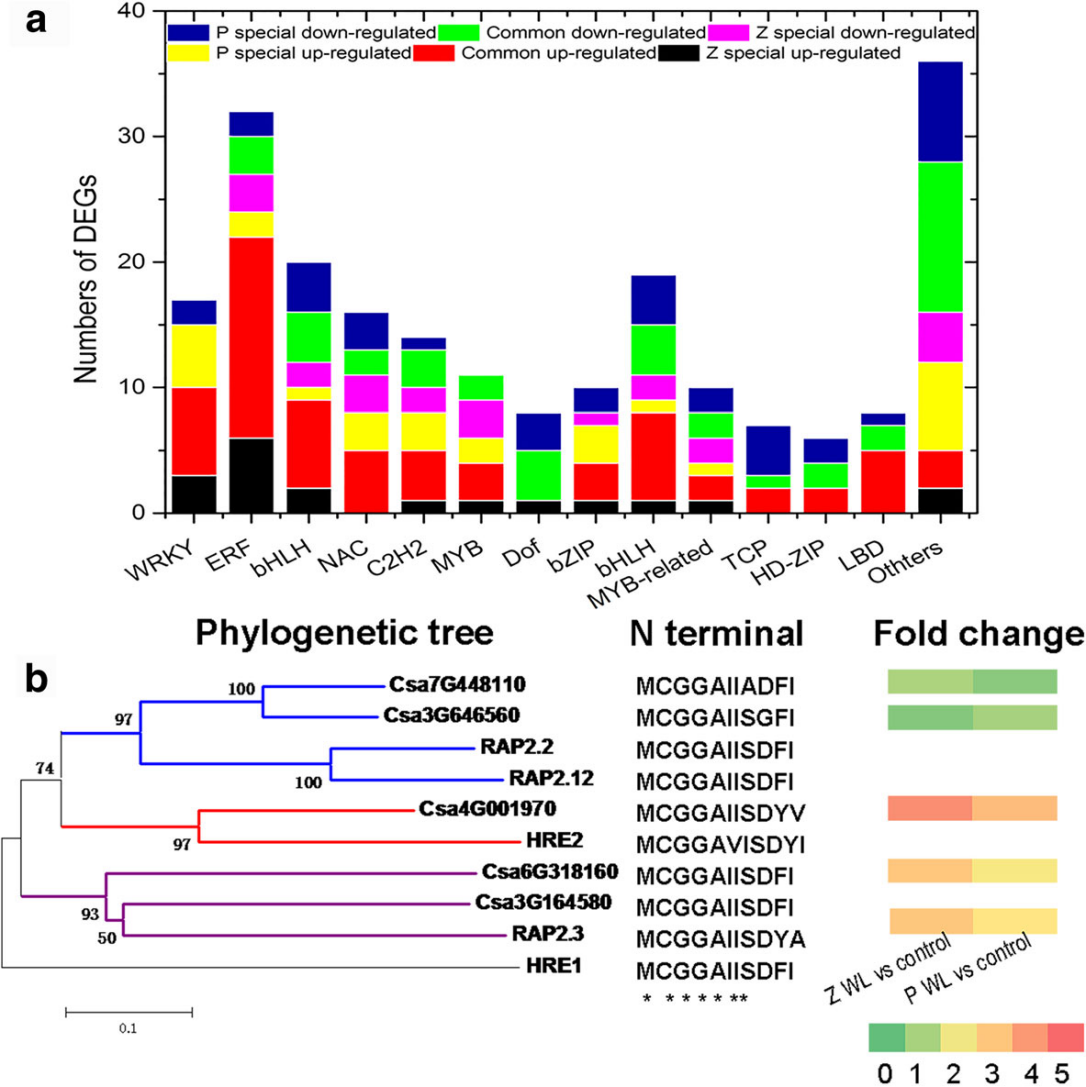
Response of transcription factors to waterlogging. **a** Graphical representations of waterlogging-regulated transcription factors based on their assigned protein families. **b** The group VII ethylene response factors in cucumber genome. The full-length protein sequences were analyzed with a neighbor-joining method. *Numbers above branches* represent the bootstrapped value from1000 replicates. *RAP2.2* (*AT3G14230*), *RAP2.12* (*AT1G53910*), *HRE2* (*AT2G47520*), *RAP2.3* (*AT3G16770*) and *HRE1* (*AT1G72360*) were Arabidopsis group VII ethylene response factors. *Asterisks* indicate a conserved motif at the N-terminus initiated with Met1-Cys2, as identified by multiple sequence alignment. ‘Z WL vs control’ represents the fold change of the gene in hypocotyls of ‘Zaoer-N’ 2 days after waterlogging treatment than unwaterlogged control; ‘P WL vs control’ represents the fold change of the gene in hypocotyls of ‘Pepino’ 2 days after waterlogging treatment than unwaterlogged control

TFs are a group of DNA-binding proteins that control target gene expression by specifically binding to cis-acting elements in their promoters and, as such, play essential roles in multiple cellular processes, such as development, cell cycle regulation, transcription regulation and responses to environmental stress [58]. In total 214 waterlogging-regulated *TFs* were identified according to their assigned gene families, which accounted for ~10% of the total DEGs (Additional file 5: Table S4). Of these, 107 TFs (66 up-regulated and 41 down-regulated) were commonly regulated in ‘Zaoer-N’ and ‘Pepino’, whereas 41 TFs (19 up-regulated and 22 down-regulated) were regulated in ‘Zaoer-N’ only, and 66 TFs (28 up-regulated and 38 down-regulated) were regulated in ‘Pepino’ only. Remarkably, *ethylene response factor* (ERFs, 32 DEGs) and *WRKYs* (17 DEGs) were included in the up-regulated group, while *DNA-binding with one finger* (*Dof*, 8 DEGs) was included in the down-regulated group (Fig. 5a). These data strongly suggested the essence of transcriptional regulation in response to waterlogging. An in-depth analysis of these waterlogging-responsive *TFs* is required to further provide candidate genes for improving waterlogging tolerance in cucumber.

Plant-specific group VII ERFs have emerged as pivotal modulators of the anaerobic responses under waterlogging conditions [59]. For example, the rice *SUBMERGENCE 1A* gene confers prolonged submergence tolerance [60]. A HMMER-based search against the cucumber genome using the AP2/ERF domain (IPR001471) and N-end motif [MCGAII(A/S)] as queries identified five group VII *ERF* genes (*Csa4G001970*, *Csa7G448110*, *Csa3G646560*, *Csa6G318160* and *Csa3G164580*) (Fig. 5b). Consistent with activated ethylene synthesis, four group VII *ERF*s were induced at higher levels in ‘Zaoer-N’ than in ‘Pepino’ (Fig. 5c), which indicates that the increased transcription of these genes could mechanistically explain hypoxic priming by ethylene [59]. *Hypoxia responsive ERF 1* (*HRE1*), *HRE2* and *RAP2.12*, three Arabidopsis ERF-VII genes, improved waterlogging tolerance by directly activating the low oxygen responsive marker gene *ADH* [61, 62]. Whether the protein-to-protein interaction effect also existed in cucumber hypocotyls when waterlogged has yet to be further validated.

### Increased cellular activity upon waterlogging

Here, the transcriptome profiling confirmed the activation of cell division during the initial stage of AR emergence in waterlogged ‘Zaoer-N’, but not ‘Pepino’, hypocotyls. In plants, A and B-type cyclins act in the G_2_-to-M transition, while D-type cyclins have been implicated in the G_1_-to-S transition [63, 64]. Four genes encoding mitotic A- and B-type cyclins (*Csa7G071600*, *Csa4G664480*, *Csa4G658580* and *Csa4G664480*) were clearly accumulated in ‘Zaoer-N’, specifically. These findings corroborate those of *Arabidopsis*, in which the cyclin-dependent kinase activity required for mitosis was regulated by redundant genes encoding CYCLINB and CYCLINA2 [63].

DEGs encoding microtubules (MTs) and MT-associated proteins, such as kinesins, had different expression levels during adventitious rooting. MTs play important roles in cell elongation and division, and they indirectly regulate morphogenesis [65]. Several genes encoding MT and MT-associated proteins, such as Csa*5G169090*, *Csa1G033010*, *Csa4G101270*, *Csa3G062600*, *Csa4G293320* and *Csa6G382990*, were highly expressed in ‘Zaoer-N’ hypocotyls only, and coincided with the activation of cell division. An increase in the ability to form AR after a subtle perturbation of the MTs has been previously shown for *Eucalyptus grandis* cuttings [66].

Furthermore, a strong induction of DEGs encoding specific histone variants (*Csa7G390230*, *Csa7G390240*, *Csa6G193590* and *Csa4G290220*) were found in ‘Zaoer-N’, whereas two tandemly arrayed DEGs encoding histone deacetylase (*Csa6G150540* and *Csa6G150550*) were induced only in ‘Pepino’. In *Arabidopsis*, the histone variant H3.3 is associated with active genes and shows a positive correlation with their expression levels, suggesting that histone variant replacement may contribute to the reprogramming at the developmental transitions in ‘Zaoer-N’ [67]. By contrast, the *Arabidopsis* histone deacetylases HDA6 and HDA19 redundantly regulate embryonic genes and negatively affect callus formation from the hypocotyls [68]. Thus, we speculated that an accumulation of histone deacetylases in ‘Pepino’ may be one of the reasons why the emergence of AR primordia was compromised.

### Other identified DEGs

Our focus on genes with significant roles in AR primordia initiation under waterlogging conditions may provide a restricted view of the large numbers of genes illuminated in this study. Therefore, in this section, other subsets of interesting DEGs are considered to provide a broader view of the molecular mechanisms involved in the waterlogging response.

First, 28 and 26 DEGs encoding cytochrome P450 (CYP) proteins in ‘Zaoer-N’ and ‘Pepino’, respectively, were identified. Le Provost et al. [69] also reported the differentially regulation of CYP proteins in the stems of oak seedlings subjected to waterlogging. The gene (*Csa5G153010*) encoding CYP86A7 showed a peculiar behavior. It was up-regulated in the tolerant genotype ‘Zaoer-N’ but dramatically down-regulated in the sensitive genotype ‘Pepino’. Even though plant CYPs contribute to the biosynthesis and/or catabolism of all phytohormones, pigments, fragrances, flavours, antioxidants, allelochemicals and defense compounds, the eight members of the *Arabidopsis* CYP86A (from 1 to 8) subfamily have been functionally defined as fatty acid ω-hydroxylases [70]. Increase in free fatty acids have been reported in waterlogged oat and cotton seedlings [71, 72]. The total level of free fatty acids might increased in waterlogged ‘Zaoer-N’ hypocotyls because of the significant accumulation of GDSL esterases/lipases, a subclass of lipolytic enzymes that can break down lipids into free fatty acids [2]. The increase in the free fatty acids was presumably due, in part at least, to the synthesis of lipids for new membranes of the AR primordia. However, increased levels of free fatty acids from lipid catabolism might also cause the disruption of membrane structures and decreased membrane stability [2]. The expression of the *CYP86A* gene may thus be involved in prevention of the redundant accumulation of the toxic levels of free fatty acids in waterlogged ‘Zaoer-N’ hypocotyls. However, the precise roles of other CYPs in the adaptation to the waterlogged conditions remain to be uncovered.

Second, 14 DEGs encoding ankyrin repeat-containing proteins were identified. Among these genes, eight DEGs (*Csa1G058080*, *Csa5G409690*, *Csa1G058090*, *Csa3G847090*, *Csa1G058110*, *Csa6G363020*, *Csa1G058660* and *Csa7G390740*) were commonly regulated in both genotypes, while the other six DEGs (*Csa3G457650*, *Csa3G696850*, *Csa6G008010*, *Csa1G058160*, *Csa2G010200* and *Csa3G822290*) were differentially regulated in ‘Pepino’ only. Du and Chye [73] reported that both of the *Arabidopsis* ankyrin repeat-containing proteins AtACBP2 and AtACBP4 interact with the group VII ERF protein RAP2.3. Prasad et al. [74] reported that another *Arabidopsis* ankyrin repeat-containing protein, XBAT32, plays an essential role in ethylene biosynthesis as a negative regulator of ACS protein abundance, and a deletion mutant of XBAT32 led to an increased ethylene production. Thus, the overrepresentation of ankyrin repeat-containing proteins transcripts in ‘Pepino’ may account for the lack of ACS gene regulation and the relatively lower production of ethylene in this genotype.

A gene (*Csa2G406690*) encoding sulfite oxidase (SO; EC 1.8.3.1) was observed to be up-regulated in ‘Zaoer-N’ hypocotyls only. SO oxidizes sulfite to sulfate and, through cytochrome c, transfers the electrons produced to the electron transport chain, allowing for the generation of ATP in oxidative phosphorylation [75]. We hypothesized that the up-regulation of the *SO* gene might benefit to energy production. Two *SOs*, *CO499223* and *CO498687*, are also up-regulated in waterlogged cotton roots [76].

## Conclusion

The present comparative RNA-seq study provides new insights into waterlogging-triggered AR primordia initiation in cucumber. The initiation of AR upon waterlogging may be explained in several different ways. First, our results indicate that ‘Zaoer-N’ maintained a more efficient carbohydrate metabolism and regeneration of ATP and NAD^+^ than the waterlogging-sensitive ‘Pepino’ to cope with energy crises imposed by waterlogging, thereby favoring greater cell differentiation. Second, ethylene was implicated as a major hormone involved in the cucumber AR primordia initiation. In light of this, and the importance of ethylene responses in the waterlogging phenotypes of other crops, group VII ethylene-responsive TFs are promising candidates for manipulating the waterlogging response. Third, several DEGs related to cell remolding and division were specifically up-regulated in the immersed ‘Zaoer-N’ hypocotyls cells, while the DEGs that negatively affected callus formation were significantly accumulated only in ‘Pepino’, coinciding with the different capabilities of the two lines to initiate de novo AR primordia. Upcoming work will aim to characterize the identified individual DEGs to understand their specific functions in waterlogging acclimation and AR formation.

## Additional files

Additional file 1: Table S1. Detailed primer sequences for qPCR confirmation. (XLS 9 kb)

Additional file 2: Figure S1. Comparison of the adventitious root numbers generated in Zaoer-N (left) and Pepino (right) hypocotyls 7 days after waterlogging treatment. (JPEG 977 kb)

Additional file 3: Table S2. Functional annotation of differentially expressed genes identified by RNA-seq in Zaoer-N hypocotyls 48 h after waterlogging treatment. ‘FC’ represents fold change of the gene. (XLS 852 kb)

Additional file 4: Table S3. Functional annotation of differentially expressed genes identified by RNA-seq in Pepino hypocotyls 48 h after waterlogging treatment. ‘FC’ represents fold change of the gene. (XLS 1012 kb)

Additional file 5: Table S4. List of differentially expressed genes annotated as transcription factors. (XLS 43 kb)

## Abbreviations

1-MCP: 1-methylcyclopropene
ABA: Abscisic acid
ACO: 1-aminocyclopropane-1-carboxylicacid oxidase
ACS: 1-aminocyclopropane-1-carboxylicacid synthase
ADH: Alcohol dehydrogenase
ARF: Auxin response factors
ARs: Adventitious roots
CYP: Cytochrome P450
DEGs: Differentially expressed genes
Dof: DNA-binding with one finger
ERFs: Ethylene Response Factor
FDR: False discovery rate
FPKM: Fragments Per Kilobase of exon model per Million mapped reads
GH3: indole-3-acetic acid-amido synthetases
GO: Gene ontology
IAA: Indole-3-acetic acid
MTs: microtubules
NCED: 9-cis epoxycarotenoid dioxygenase
NsHBs: Non-symbiotic hemoglobins
NTRs: nitrate transporters
PDC: Pyruvate decarboxylase
PIN: Auxin efflux carrier components
QPCR: Quantitative PCR
ROS: Reactive oxygen species
SO: Sulfite oxidase
TFs: Transcription factors
YUC: indole-3-pyruvate monooxygenase YUCCA
