# Supplementary figures and images for: Comparative RNA-seq based transcriptome profiling of waterlogging response in cucumber hypocotyls reveals novel insights into the de novo adventitious root primordia initiation
Source: BMC Plant Biol. 2017 Jul 26;17:129. doi: 10.1186/s12870-017-1081-8 (PMC5530484; doi:10.1186/s12870-017-1081-8)

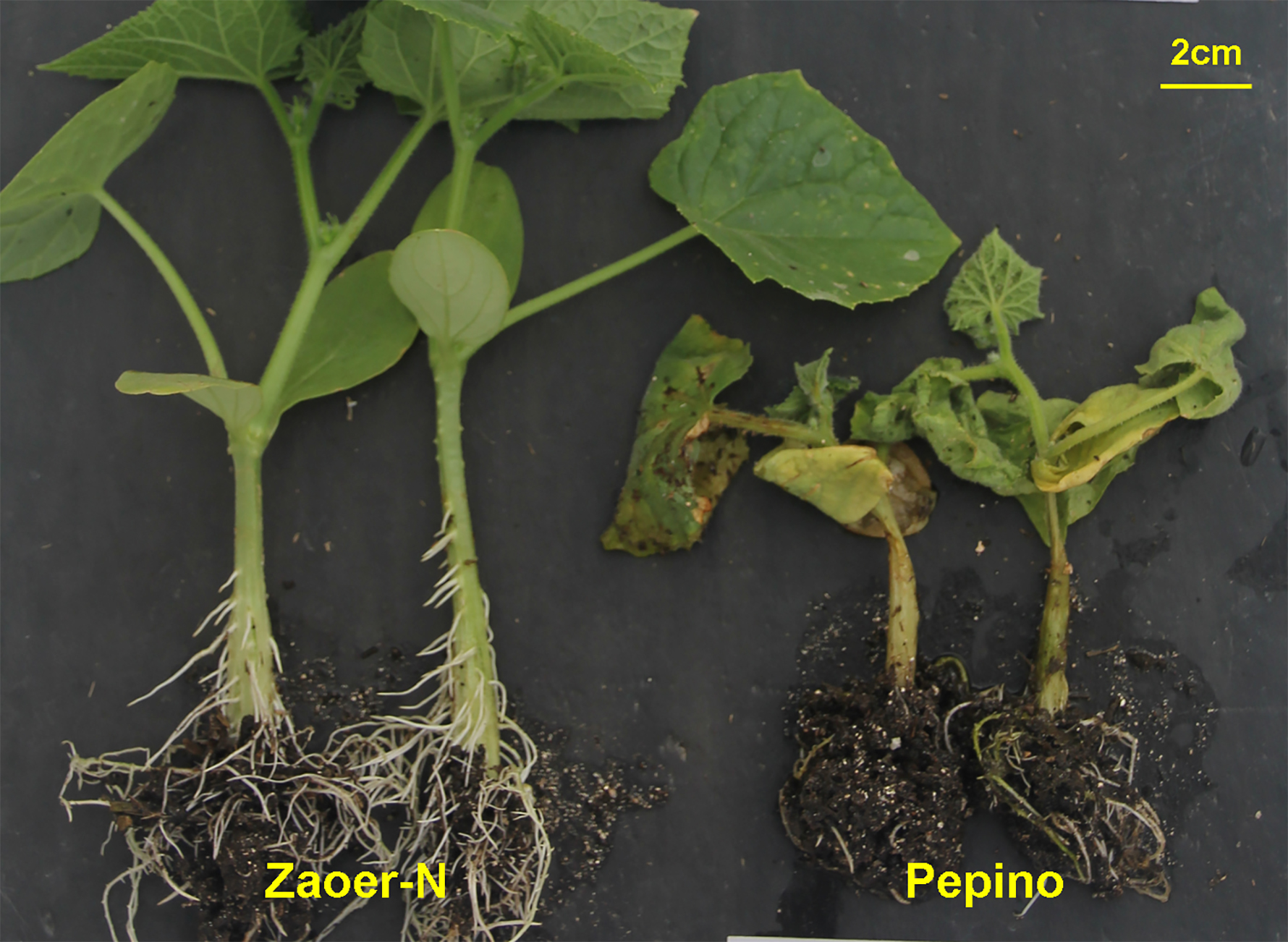

Supplement: Supplementary file 2 — Comparison of the adventitious root numbers generated in Zaoer-N (left) and Pepino (right) hypocotyls 7 days after waterlogging treatment. (JPEG 977 kb) [file 12870_2017_1081_MOESM2_ESM.jpg]
